# Exploratory spatial analysis of Lyme disease in Texas –what can we learn from the reported cases?

**DOI:** 10.1186/s12889-015-2286-0

**Published:** 2015-09-19

**Authors:** Barbara Szonyi, Indumathi Srinath, Maria Esteve-Gassent, Blanca Lupiani, Renata Ivanek

**Affiliations:** International Livestock Research Institute (ILRI), Box 5689, Addis Ababa, Ethiopia; Center for Agribusiness Excellence, Tarleton State University, Stephenville, TX USA; Department of Veterinary Pathobiology, College of Veterinary Medicine and Biomedical Sciences, Texas A&M University, College Station, TX USA; Department of Veterinary Integrative Biosciences, College of Veterinary Medicine and Biomedical Sciences, Texas A&M University, College Station, TX USA

**Keywords:** *Borrelia burgdorferi*, Exploratory spatial data analysis, Lyme disease, Texas

## Abstract

**Background:**

Lyme disease (LD) is a tick-borne zoonotic illness caused by the bacterium *Borrelia burgdorferi*. Texas is considered a non-endemic state for LD and the spatial distribution of the state’s reported LD cases is unknown.

**Methods:**

We analyzed human LD cases reported to the Texas Department of State Health Services (TX-DSHS) between 2000 and 2011 using exploratory spatial analysis with the objective to investigate the spatial patterns of LD in Texas. Case data were aggregated at the county level, and census data were used as the population at risk. Empirical Bayesian smoothing was performed to stabilize the variance. Global Moran’s I was calculated to assess the presence and type of spatial autocorrelation. Local Indicator of Spatial Association (LISA) was used to determine the location of spatial clusters and outliers.

**Results and Discussion:**

There was significant positive spatial autocorrelation of LD incidence in Texas with Moran’s I of 0.41 (p = 0.001). LISA revealed significant variation in the spatial distribution of human LD in Texas. First, we identified a high-risk cluster in Central Texas, in a region that is thought to be beyond the geographical range of the main vector, *Ixodes scapularis*. Second, the eastern part of Texas, which is thought to provide the most suitable habitat for *I. scapularis*, did not appear to be a high-risk area. Third, LD cases were reported from several counties in western Texas, a region considered unsuitable for the survival of *Ixodes* ticks.

**Conclusions:**

These results emphasize the need for follow-up investigations to determine whether the identified spatial pattern is due to: clustering of misdiagnosed cases, clustering of patients with an out-of state travel history, or presence of a clustered unknown enzootic cycle of *B. burgdorferi* in Texas. This would enable an improved surveillance and reporting of LD in Texas.

**Electronic supplementary material:**

The online version of this article (doi:10.1186/s12889-015-2286-0) contains supplementary material, which is available to authorized users.

## Background

Lyme disease (LD) is the most frequently reported vector-borne disease in the United States with more than 30,000 cases reported annually to the Centers for Disease Control and Prevention (CDC) [1]. Typical symptoms of LD in humans include fever, headache, fatigue, and a characteristic skin rash called *erythema migrans*. If left untreated, late LD usually manifests as chronic arthritis, but less commonly can also include the nervous system (meningitis, facial palsy, rarely encephalitis), the heart (conduction and rhythm disturbances, myocarditis) and the eyes (conjunctivitis, uveitis) [2].

Lyme disease is maintained in enzootic cycles using arthropod vectors and vertebrate reservoir hosts. The causative agent of LD in North America, *Borrelia burgdorferi* sensu stricto occurs naturally in a complex enzootic cycle in which the white-footed mouse (*Peromyscus leucopus*) acts as a reservoir together with other small mammals including squirrels, chipmunks and shrews. *Ixodes scapularis*, the main competent tick vector for the transmission of this pathogen in the United States, becomes infected with *B. burgdorferi* while feeding on the reservoir hosts. During subsequent blood meals, the *Ixodes* ticks can transmit infection among reservoir hosts or to incidental hosts, including humans [3, 4]. Lyme borreliosis is emerging in the United States mostly due to the current invasive spread of *I. scapularis* from endemic foci in the Northeast and Upper Midwest to previously non-endemic areas [5]. Understanding spatial patterns of LD is crucial to effectively target intervention strategies, allocate resources and raise awareness in high-risk areas.

There has been much controversy over the presence and extent of LD in Texas in recent years, involving the scientific community and the public media. Adding to the confusion is that another condition, namely the Southern Tick-Associated Rash Illness (STARI), also called “Lyme-like disease”, presents with Lyme-like symptoms including the *erythema migrans* rash [6]. The natural history and etiology of STARI is still unknown, and the existence and severity of symptoms other than the presence of the *erythema migrans* rash are uncertain (http://www.cdc.gov/stari//; Accessed: July 6, 2015). STARI is thought to be caused by the bite of the Lone Star tick (*Amblyomma americana)* which is found throughout the southeastern and south-central United States, and south-central United States, and it is the most common tick biting humans in these areas. [6]. However, there is no surveillance for STARI and its incidence is unknown [6]. Confusion exists between LD and STARI in diagnosis and reporting, because STARI meets the surveillance definition of LD, and doctors sometimes report STARI patients as having LD [6]. Although LD is uncommon and traditionally non-endemic in Texas, the vector tick for LD does exist in Texas and infection with *B. burgdorferi* has been documented in the state [7–11]. Also, Texas reports 50–100 cases of LD to the CDC every year [1]. Currently, the status of LD in Texas is not clear, and spatial analysis of reported cases can provide valuable public health information. Lyme borreliosis is a reportable disease, and cases of LD in Texas are reported to the Texas Department of State Health Services (TX DSHS). The goal of this study was therefore to conduct exploratory spatial analysis of human LD cases reported to the TX DSHS.

## Methods

### Study area

Located in the South Central United States, Texas is the largest state in the 48 contiguous United States with a growing population of over 25 million residents. The population is not evenly distributed throughout the state, with approximately a third of the population living in metropolitan areas in the eastern part of the state (United States Census Bureau, www.census.gov; Accessed: Nov 23, 2012). Due to the large size of Texas, it has multiple climate zones and ecological regions, each with unique vegetation and wildlife. Texas also has wide variations in precipitation patterns with eastern Texas having substantially higher precipitation compared to the western half of the state, where extremely dry conditions often prevail [12].

### Data sources

A computerized dataset consisting of all LD cases reported to the TX DSHS between 01/01/2000 and 12/31/2011 was obtained for analysis. The case definition criteria used by the TX DSHS follows that of the CDC definition of a LD case, which is currently defined as a person with (1) *erythema migrans* with known exposure or (2) *erythema migrans* with laboratory confirmation of infection and without a known exposure or (3) at least one late manifestation and laboratory confirmation of infection. Laboratory confirmation consists of (i) positive culture for *B. burgdorferi* or (ii) positive enzyme immunoassay (EIA) or immunofluorescent assay (IFA) followed by a Western immunoblot or (iii) positive IgG immunoblot only or (iv) cerebrospinal fluid antibody positive for *B. burgdorferi* by EIA or IFA, when the titer is higher than it was in serum. (http://wwwn.cdc.gov/nndss/conditions/lyme-disease/case-definition/2011/; Accessed: June 30, 2015). If a person meets any one of these 3 criteria, they meet the surveillance criteria for LD, and therefore are reported as a LD case.

Texas county shapefile and demographic files were obtained from the publicly available Texas Natural Resources Information System website (https://tnris.org; Accessed: October 15, 2012). Spatial data were projected to Albers equal-area conic projection, North American Datum 1983 (NAD83). Census data were used as the population at risk in calculating incidence, which was defined as the number of reported cases within a defined time frame and area divided by the population at risk. Cumulative incidence for each county was calculated as all reported cases over the study period (2000–2011) divided by the total population in the respective county. In order to obtain a more accurate incidence estimate for LD in Texas over the study period, incidence estimates were calculated by taking into account the increasing population at risk in the state as follows: for the years 2000–2003, the 2000 census data were used; for 2004–2007, the average of the 2000 and 2010 census data were used; and for years 2008–2011, the 2010 census data were used as the denominator. A shapefile (NAD83, Albers Equal Area Conic) with Level III eco-regions in Texas was obtained from the publicly available website of the Western Ecology Division of United States Environmental Protection Agency (http://www.epa.gov/wed/pages/ecoregions/tx_eco.htm; Accessed June 22, 2015) and mapped to visualize the locations of the identified clusters.

### Spatial smoothing using empirical Bayesian approach

Crude disease incidence estimates can be unstable in areas where the sampled population is small and/or the population at risk varies considerably, which can produce bias and spurious outliers [13]. In Texas, the county level population (population at risk) varies substantially, and several counties had few or no reported LD cases during the study period. Therefore, it was necessary to smooth the crude incidence estimates to address this variance instability. The smoother technique implemented in this study was Empirical Bayesian smoothing, in which the crude incidence for each county was shrunk towards the overall average incidence for the entire study area [14].

### Assessment of spatial autocorrelation

Spatial autocorrelation refers to the correlation of a variable, in this instance incidence of LD, with itself in space. Positive spatial autocorrelation exists if high incidence of LD correlates with high incidence in neighboring counties (“hot spots”) or when low incidence correlates with low incidence in neighboring counties (“cold spots”). Negative spatial autocorrelation exists in the data if high incidence correlates with low incidence in neighboring counties and vice versa (spatial outliers) [15].

In order to assess the extent of similarity between locations and values, a neighborhood structure was imposed on the data using a weights matrix. A contiguity-based spatial weight matrix using rook structure was constructed, where counties that share a common border were considered neighbors [16]. Spatial autocorrelation analysis requires constant variance, which was violated because the crude incidence of LD varied greatly from county to county. Therefore, empirical Bayesian smoothed incidence estimates were used to assess spatial autocorrelation. To determine the significance of spatial clustering, a permutation test was conducted and pseudo-p values were calculated by comparing the observed spatial distributions to spatially randomized reference distributions [15]. Two types of spatial autocorrelation analyses were performed as follows: global spatial autocorrelation was measured by Global Moran’s I, while local spatial autocorrelation was measured using Local Indicators of Spatial Association (LISA). Moran's I was carried out to capture the extent of overall clustering of LD in Texas and to evaluate whether the spatial pattern was clustered, dispersed, or random. Moran’s I ranges from −1 to 1 with a positive value indicating tendency toward clustering, while a negative value indicates tendency toward dispersion. A Moran scatter plot was also produced to examine county incidence in relation to their neighbors (Additional file 1) [17].

While Moran’s I characterizes the type and strength of spatial autocorrelation in the data, overall, it does not indicate the actual location of significant spatial clusters and outliers. For this purpose, a LISA cluster map was produced that classifies counties based on the type of spatial association. The high-high and low-low locations are indications of spatial clusters (hot and cold spots respectively) while low-high and high-low associations are indications of spatial outliers [18]. Different significance filters (0.05, 0.01, 0.001) and permutations (99, 199, 999) were applied to assess the sensitivity of the results and ensure the stability of reported clusters and outliers. The final LISA map was based on 999 permutations and a pseudo-significance level of *p* = 0.05 (Anselin [16]).

## Results

**Fig. 1:**
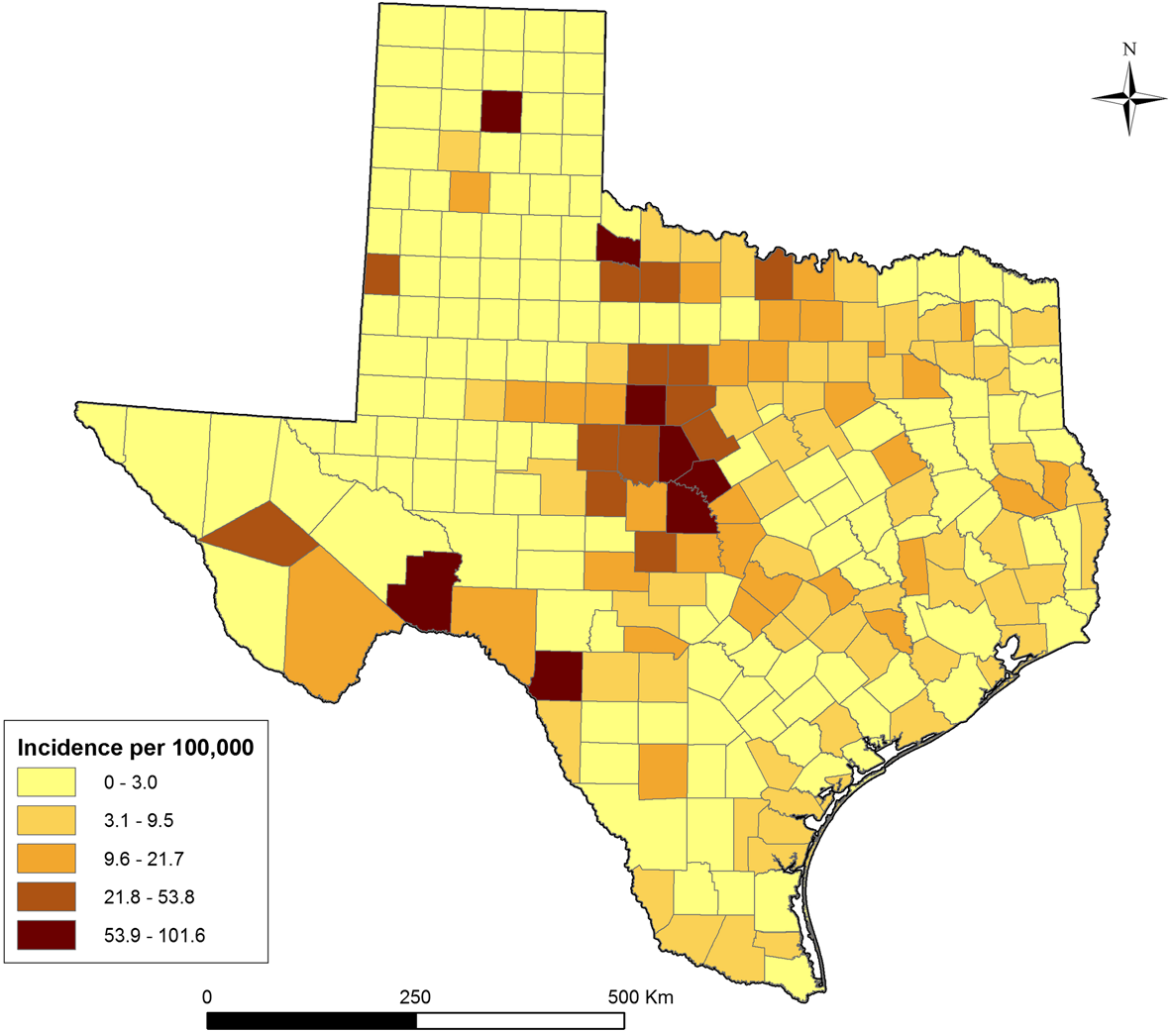
Choropleth map showing crude cumulative Lyme disease incidence for the period from 2000 to 2011 in Texas.

**Fig. 2:**
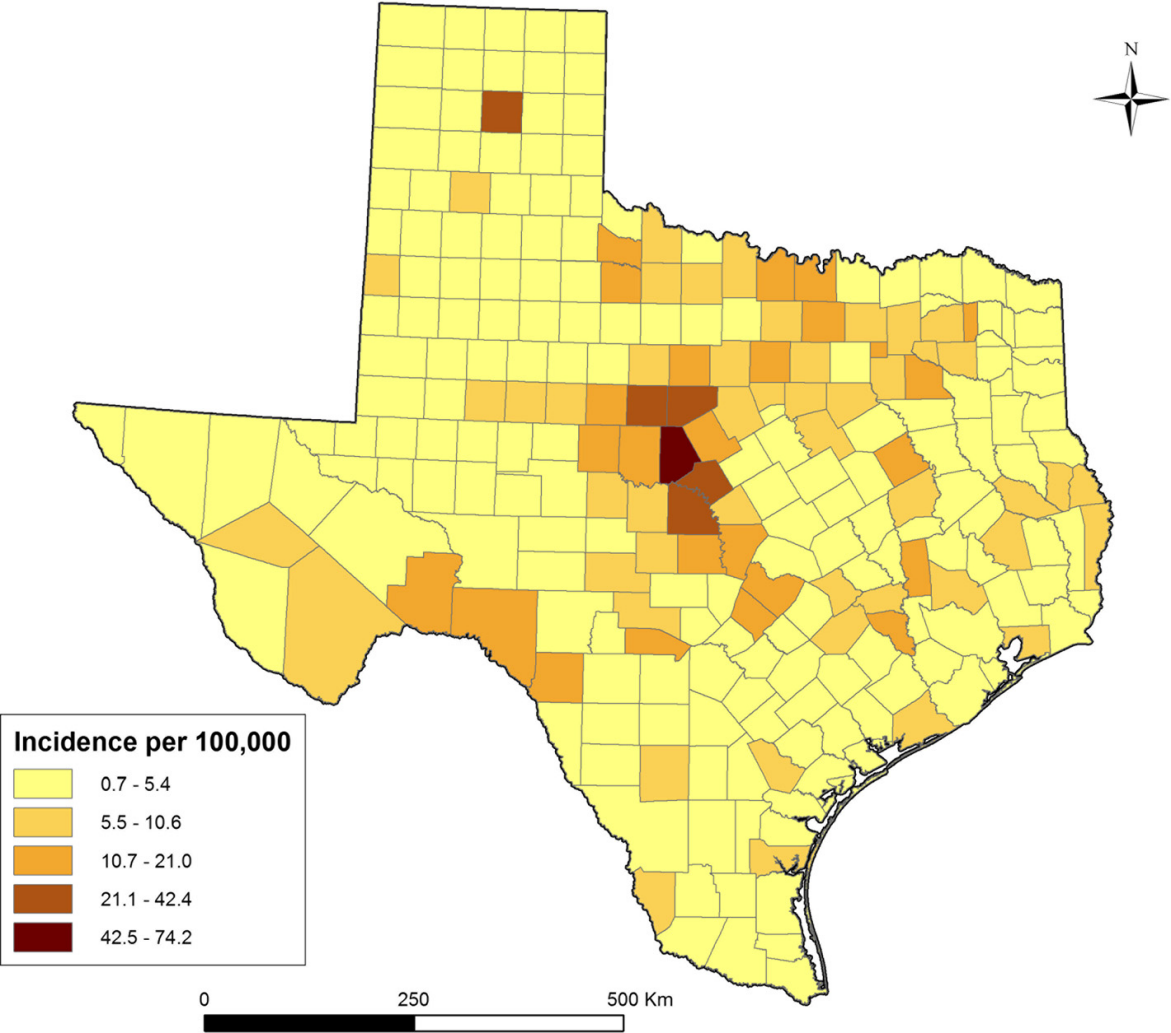
Choropleth map showing spatially smoothed Lyme disease incidence in the period from 2000 to 2011 in Texas based on the empirical Bayesian smoothing.

There were a total of 1,286 reported LD cases in Texas between 01/01/2000 and 12/31/2011. The number of reported LD cases ranged between 29 and 276 per year. Since 2000 the annual state-wide incidence typically has varied between 0.4-0.6 cases per 100,000 population. Over half of all Texas counties (140/254) reported at least one case during the study period. Approximately 40 % of all cases were reported from the metropolitan areas of Austin, Houston, and Dallas. Figure 1 shows the geographic distribution of crude county-level cumulative LD incidence in Texas over the study period. There were 8 counties where the cumulative incidence was higher than 60 cases per 100,000 population. These counties were located in Central (Brown, San Saba, Callahan, Mills) Northern (Carson, Foard), and Southwestern (Terrel, Kinney) Texas. As expected, empirical Bayesian smoothing resulted in reduced variation of the cumulative incidence estimates (Fig. 2). Bayesian smoothing adjusted the incidence considerably in counties where the population at risk was smaller, most notably in the southwestern counties. Even after smoothing, the adjusted incidence in Central Texas remained the highest (Brown, Callahan, and Eastland counties). Low population density in the western counties might have created spurious results in the crude incidence map.

**Fig. 3:**
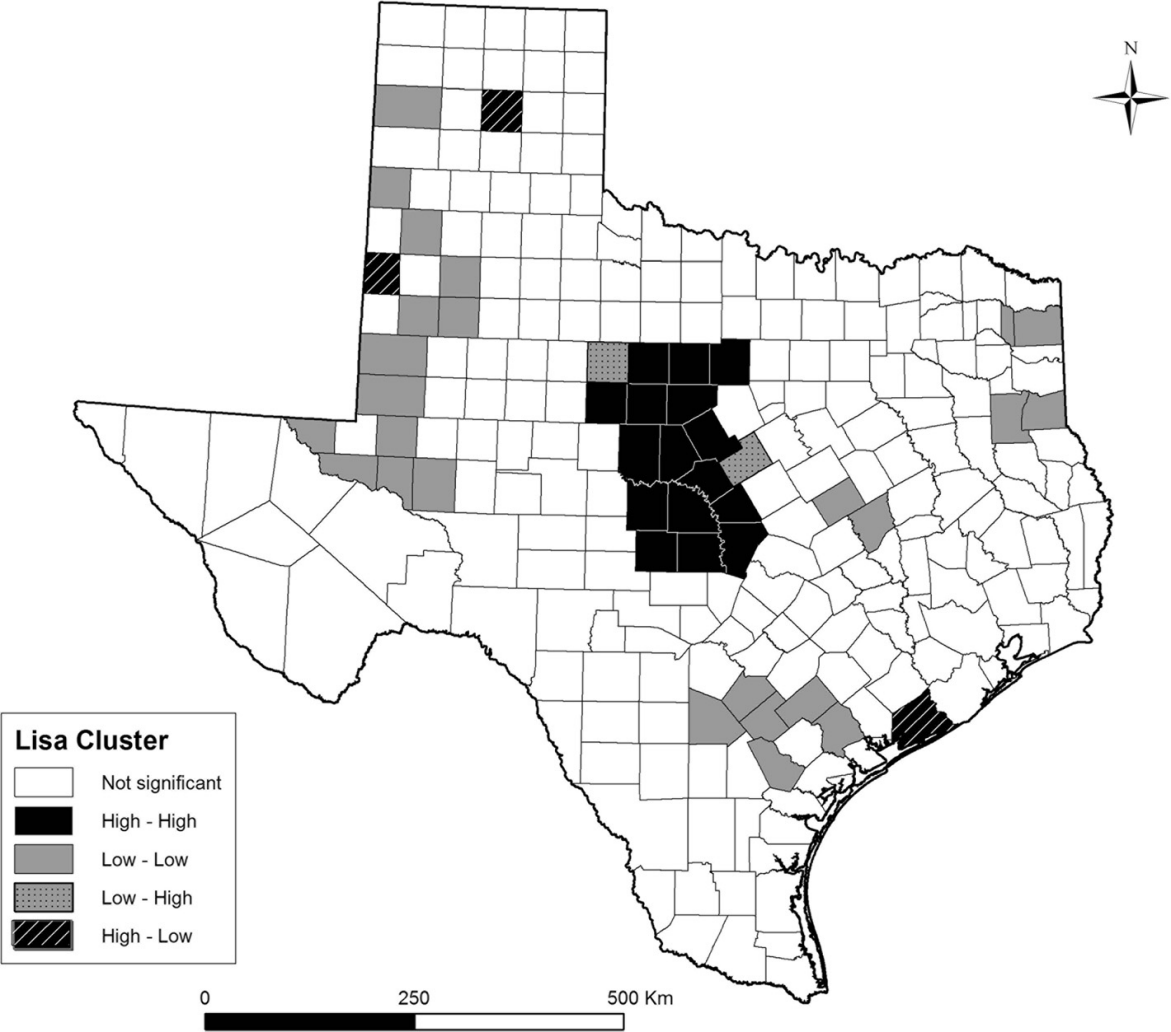
Cluster map of Local Indicators of Spatial Association (LISA) of Lyme disease in Texas based on the empirical Bayesian smoothed incidence estimates. The high-high and low-low locations are spatial clusters (hot and cold spots, respectively) while low-high and high-low associations are spatial outliers. A significance level of 0.05 was used.

**Fig. 4:**
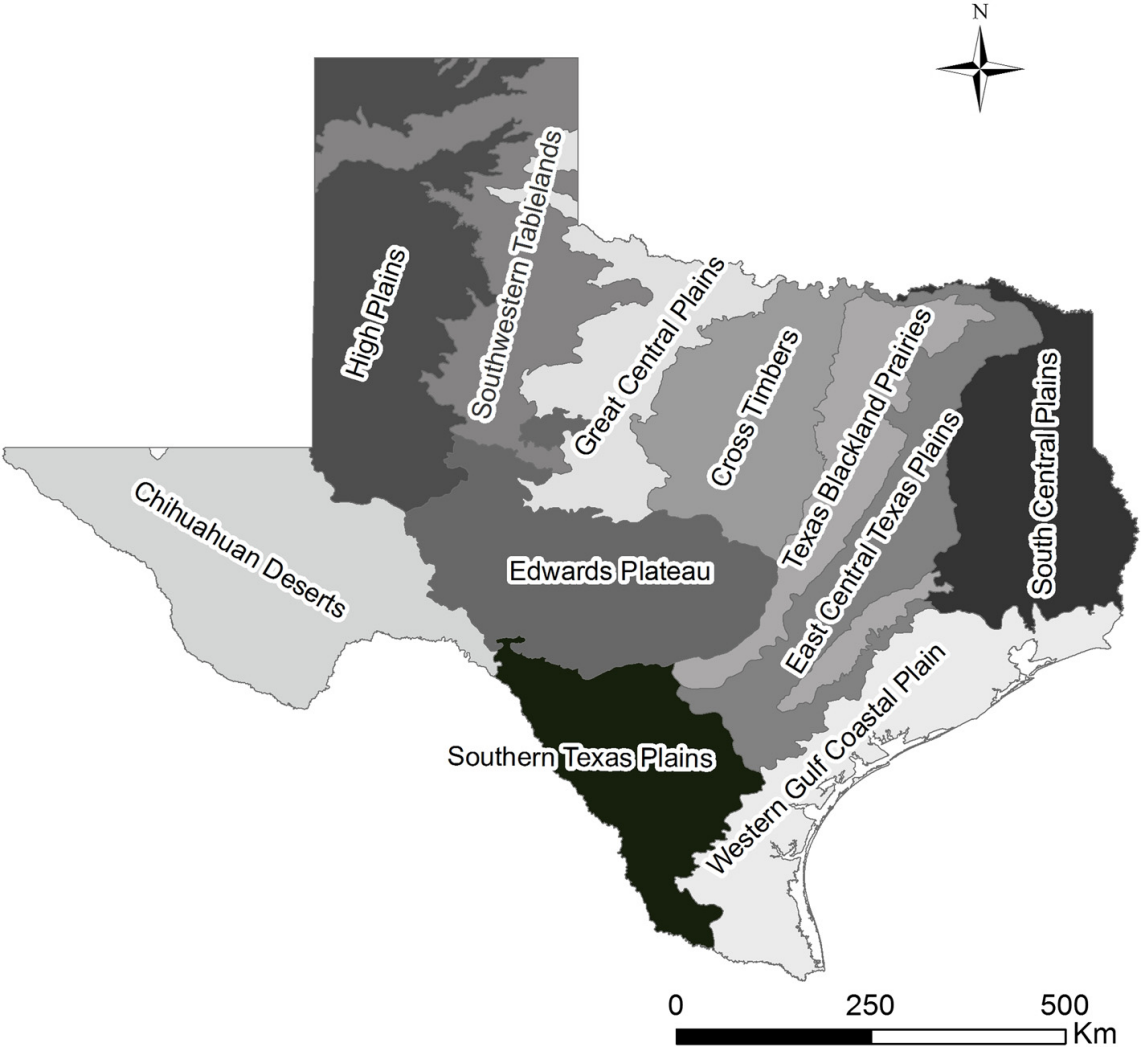
Map of eco-regions (level III) in Texas.

There was a significant positive spatial autocorrelation of LD incidence in Texas with Global Moran’s statistics of 0.4155 (pseudo *p* = 0.001) based on empirical Bayesian smoothed rates. Figure 3 shows the LISA cluster map depicting the types of spatial autocorrelation between counties with LD cases. The high-high clusters were concentrated in Central Texas while low-low clusters occurred in different regions, especially the Low Plains region in the Panhandle. Several spatial outliers were also detected. There were two counties with low incidence surrounded by counties with high incidence located in Central Texas (Jones and Hamilton). Conversely, counties with high incidence surrounded by counties with low incidence included Cochran and Carson counties in the Panhandle and Matagorda county in the Southeast. Figure 4 shows the distribution of different eco-regions in Texas, indicating that the main high-risk area identified in this study is located in the Cross-Timbers eco-region.

## Discussion

This was the first study that examined the geographic distribution of reported human LD cases in Texas using exploratory spatial data analysis. We detected significant variation in the spatial distribution of reported cases in the state, and some illuminating patterns emerged. First, after adjusting for the population at risk, we delineated a high-risk cluster in Central Texas corresponding to the Cross-Timbers eco-region, which is a region that is considered to be beyond the geographical range of the main vector, *Ixodes scapularis* [19, 20]. Second, the eastern part of Texas, which is considered to provide the most suitable habitat for *I. scapularis*, did not appear to be a high-risk area. Third, LD cases were reported from several counties in western Texas, a region considered unsuitable for the survival of *Ixodes* ticks. There are a number of alternative explanations for the observed pattern of reported cases, namely 1) clustered misdiagnosis of cases, 2) clustered infections that were acquired elsewhere but were reported in the patients’ county of residence and 3) clustered local populations of infected, vector-competent and human-biting ticks in the high incidence counties. The significance and implications of these findings for public health, and the interpretation of the ongoing surveillance and reporting activities in the state for these possibilities, are outlined below.

The study was based on LD cases as they were reported to the public health system. It is possible, therefore, that surveillance case definitions were interpreted differently in different jurisdictions due to unequal knowledge and practices of physicians, resulting in regional over- or under-reporting bias. For instance, an *erythema migrans* rash without rigorous laboratory evidence might be reported as a LD case, even though it may not actually be LD. This can happen because STARI meets the surveillance definition of LD, and doctors are required by law to report patients with *erythema migrans* rash as having LD, which could contribute to over-reporting of LD [6]. This is particularly problematic in the southern United States, including Texas, where STARI is thought to be more common than LD, due to the abundance and aggressive biting behavior of Lone Star ticks in this state [6]. At the same time, physicians in non-endemic states may be told that there is no LD disease in their area, and that any LD diagnosis (whether true LD or misdiagnosed STARI) is in error. This might confuse physicians and they may question the importance of reporting a disease that is not officially recognized to exist in their state [6]. This in turn, could lead to underreporting of both STARI and LD*.* The findings of this study highlight the need to revise the surveillance definition for LD, and to clarify whether LD cases reported in Texas are true cases or STARI cases. If indeed most reported LD cases have been misdiagnosed (and are in fact false positives), then the surveillance and case definition criteria of LD should be carefully revised in order to assist future epidemiologic investigations, and to improve targeting of interventions to truly high-risk areas. Also, more research is needed to better understand the distribution and etiology of STARI.

The current study identified several low-high and high-low outlier counties where incidence of reported LD sharply differed from the surrounding counties. Also, the eastern part of Texas did not appear to be a high-risk area for LD. It would be of high interest to compare the diagnostic and reporting criteria for LD between the eastern part of Texas and the Cross-Timbers cluster identified in the current study, and also between the outlier and the surrounding counties, to assure that diagnostic and reporting criteria are uniformly applied across the whole state of Texas and so to maximize the benefits of the ongoing surveillance activities.

Regional epidemiologic patterns of human LD are influenced both by the heterogeneity of ecological risk factors and also by human risk behavior, particularly in terms of traveling to, and outdoors activities (such as hunting) in areas with endemic LD. There was no information about traveling history for the cases analyzed in this study to further explore this risk factor. Presumably, not all cases that were diagnosed and reported in Texas had been exposed to infected ticks in Texas. At the same time, it seems reasonable to assume that cases living in metropolitan areas were more likely to have been exposed far away from their home area, compared to cases reported in the remote areas where out-of-state travel to endemic areas may not be as likely or easily accessible. Interestingly, however, the high-risk cluster in Central Texas was located away from any metropolitan areas. Also, there are no known endemic LD areas in the neighboring states. Although this finding is intriguing, without the knowledge of travel history, no conclusions can be drawn about the place of exposure. This highlights the importance of systematic collection of information about the location of exposure of LD cases whenever known (e.g., outside vs. inside of the state) to enhance LD surveillance in Texas. In the event that the travel history and misdiagnosis (and the resulting under- and/or over-reporting bias) could not explain the observed patterns in human infection, then the pattern reflects either an advance of the tick-reservoir cycle from eastern deciduous forests, or long-distance dispersal of infected *I. scapularis* by birds [21]. Given that the eastern part of Texas did not appear to be a high-risk area for LD, local advance from the east is less likely but still possible. It might also be possible that long distance dispersal via birds could have established a new disease focus in the Cross-Timbers region, initiating a spatial cluster of infection. In the absence of *I. scapularis*, there may be alternative tick vectors (for example, *Ixodes affinis,* a competent vector of *B. burgdorferi* sensu stricto in some parts of the southern United States [22]) and host species involved in the maintenance of enzootic cycles of *B. burgdorferi* that underlies the observed patterns of human infection [5]. Currently, there is no tick surveillance in Texas which could provide insights into the distribution and possible expansion of *I. scapularis*, or into the presence of alternative tick vectors for *B. burgdorferi* in the state. Also, little is known about the vector specificity of *B. burgdorferi* to certain species of ticks. For example, in Europe, *Ixodes ricinus* in a competent vector, but the other common ticks are not. Sampling of potential vector ticks and reservoir hosts at targeted sites would be needed to determine if alternative species of vectors and reservoir hosts exist and maintain *B. burgdorferi* in Texas*.*

The Cross-Timbers is a North American eco-region that stretches from southern Oklahoma into Central Texas, forming a transitional area between the eastern deciduous forests and the grasslands of the southern Great Plains. The Cross-Timbers ecosystem is a vast mosaic of forest, woodland, savannah and prairie. The dominant trees are post oak and blackjack oak with an understory of shrubs and grasses. The variety of ecosystems in the Cross-Timbers provide diverse habitats for wildlife, and the abundant acorns are a staple food source for wild turkeys, prairie chickens, raccoons, squirrels, and deer [12]. As such, this ecoregion may well provide suitable habitat for a variety of potential host and vector species for *B. burgdorferi* and this area thus merits further investigation.

While the Cross-Timbers may provide a suitable habitat for a range of potential tick vector and mammalian reservoir host species for *B. burgdorferi*, the arid conditions and sparse vegetation in West Texas limits the survival of several species that inhabit the eastern half of the state. It has been suggested that, in parts of the southern United States, host composition may be dominated by non-mammalian species such as lizards [23]. However, the role of lizards in maintaining *B. burgdorferi* is unknown. It is also unclear which tick species might serve as vectors in these arid ecosystems. More studies are needed to shed light on the presence of an enzootic cycle of this pathogen in the southern United States.

In recent years, the annual incidence of LD in Texas has typically varied between 0.4-0.6 cases per 100,000 population. To put these numbers into perspective, a national study of 15-year duration (1992–2006) revealed mean annual cumulative incidence of LD for all states to have ranged from <0.01 cases per 100,000 in Montana and Colorado to 73.6 per 100,000 cases in the highly endemic state of Connecticut, with a median of 0.5 cases per 100,000 population [24]. Thus, the reported incidence of LD in Texas typically has been around the national median rate.

Some limitations to this study exist. While exploratory spatial data analysis is particularly suitable for visualizing and exploring spatial data and for detecting interesting spatial patterns, an inherent limitation of this method is that it does not explain the patterns it reveals [15]. Thus, as discussed above, further studies are needed to explain the spatial patterns we observed and to determine the specific anthropogenic, environmental and ecological factors responsible for distribution of LD in Texas.

## Conclusions

In conclusion, while LD is not considered endemic in Texas, cases have been reported throughout Texas within the past decade. We identified a high-risk cluster of reported LD cases in Central Texas, in the Cross-Timbers eco-region. In contrast, the eastern part of Texas, which has been thought to provide the most suitable habitat for the main tick vector, did not appear to be a high-risk area. The results and their possible explanations in terms of the possible role of misdiagnosis and travel history highlight the need for improved surveillance and case definition criteria for LD. Further studies need to be done to determine if alternative host and vector species may be involved in the maintenance of enzootic cycles of *B. burgdorferi* in Texas.

## Additional file

Additional file 1:
**Moran’s I scatter plot of smoothed Lyme disease incidence in Texas counties, 2000–2011.** The slope of the scatter plot corresponds to the value for Moran's I. The four quadrants of the scatter plot visualize the type and strength of spatial autocorrelation among neighboring counties, namely high-high, low-low (positive spatial autocorrelation) and high-low, low-high (negative spatial autocorrelation). (TIFF 409 kb)

## Abbreviations

CDC: Centers for disease control and prevention
LD: Lyme disease
LISA: Local Indicators of Spatial Association
STARI: Southern tick associated illness
TX DHSH: Texas Department of State Health Services
