# Supplementary figures and images for: Exploratory spatial analysis of Lyme disease in Texas –what can we learn from the reported cases?
Source: BMC Public Health. 2015 Sep 19;15:924. doi: 10.1186/s12889-015-2286-0 (PMC4575478; doi:10.1186/s12889-015-2286-0)

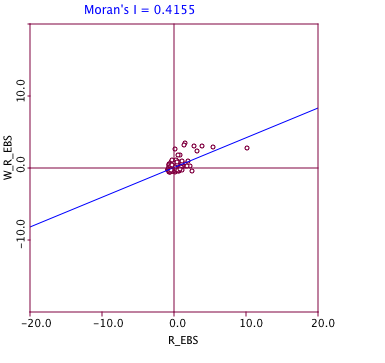

Supplement: Additional file 1: — Moran’s I scatter plot of smoothed Lyme disease incidence in Texas counties, 2000–2011. The slope of the scatter plot corresponds to the value for Moran's I. The four quadrants of the scatter plot visualize the type and strength of spatial autocorrelation among neighboring counties, namely high-high, low-low (positive spatial autocorrelation) and high-low, low-high (negative spatial autocorrelation). (TIFF 409 kb) [file 12889_2015_2286_MOESM1_ESM.tiff]
